# Supplementary material for: In vivo inducible reverse genetics in patients’ tumors to identify individual therapeutic targets
Source: Nat Commun. 2021 Sep 27;12:5655. doi: 10.1038/s41467-021-25963-z (PMC8476619; doi:10.1038/s41467-021-25963-z)
Supplement: Supplementary file 1 — Supplementary Information [file 41467_2021_25963_MOESM1_ESM.pdf]

# Supplementary information

## In vivo inducible reverse genetics in patients' tumors to identify individual therapeutic targets

Michela Carlet[1*], Kerstin Völse[1*], Jenny Vergalli[1], Martin Becker[1], Tobias Herold[1,2,3], Anja Arner [4], Daniela Senft[1], Vindi Jurinovic[1,2,4], Wen-Hsin Liu[1], Yuqiao Gao[1], Veronika Dill[5], Boris Fehse[6], Claudia D. Baldus[7], Lorenz Bastian[7], Lennart Lenk[8], Denis M. Schewe[8], Johannes W. Bagnoli [9], Binje Vick[1], Jan-Phillip Schmidt[1], Alexander Wilhelm[10], Rolf Marschalek[10], Philipp J. Jost[3,5,11], Cornelius Miething[12], Kristoffer Riecken[6], Marc Schmidt-Supprian[13], Vera Binder[4], Irmela Jeremias[1,3,4, ☆]

<u>This file contains</u>:

Supplementary Tables

Supplementary Figures and Legends

# Supplementary Tables

## Supplementary Table 1. Clinical characteristics of AML and ALL patients

| sample | disease stage* | age | cytogenetics | mutations∞ | mean passaging time§ [days] | Reference |
|---|---|---|---|---|---|---|
| **AML-388** | ID | adult | *KMT2A/AFDN* | *KRAS, CEBPZ* | 47 | 1, 2 |
| **ALL-199** | R2 | child | somatic trisomy21; leukemic homozygous 9p deletion; *P2RY8-CRLF2* | N.D. | 42 | 3, 4 |
| **ALL-265** | R1 | child | hyperdiploidy with additional 6,13,14,17,18,21,X chromosome | *KMT2D, HERC1, CSMD1, PRRT2* | 43 | 3, 4 |
| **ALL-707** | ID | child | t(4;11) *KMT2A/AFF1* | N.D. | 50 | 4 |
| **ALL-811** | R1 | adult | *DUX4-IGH* | *KMT2D* | 80 | |

*when the primary sample was obtained; ∞ mutations determined by panel sequencing; §time of passaging through mice refers to the time from injection of the sample until mice had to be sacrificed due to end stage leukemia; ID = initial diagnosis; R1 = 1st relapse; R2 = 2nd relapse; f = female; m = male; N.D. not determined.


[1] Vick et al., PLoS One 2015
[2] Ebinger et al., Hematologica 2020
[3] Ebinger et al., Cancer Cell 2016
[4] Heckl et al., Leuk Lymphoma 2019

## Supplementary Table 2. Generation of transgenic PDX

| sample | mean passaging time§ [days] | Number of sortings * | Transduction efficiency [%] | | | |
|---|---|---|---|---|---|---|
| | | | Cre$^{ERT2}$ | shCTRL-iRFP | shCTRL-BFP | shGOI |
| **AML-388** | 47 | 2 | 28.5 | 20.8 | 37.9 | 30.5 |
| **ALL-199** | 42 | 2 | 9.6 | 23 | 37 | 33 |
| **ALL-265** | 43 | 2 | 22.3 | 20.5 | 41 | 28 |
| **ALL-707** | 50 | 3 | 3.39 | 2.3 | 4.7 | 3.3 |
| **ALL-811** | 80 | 2 | 0.67 | 1.75 | 26.5 | 10.8 |

§time of passaging through mice refers to the time from injection of the sample until mice had to be sacrificed due to end stage leukemia; *from a blank sample to a double-transgenic (Cre and shmiR) sample

## Supplementary Table 3. shRNA sequences

| target | guide - 22mer |
|---|---|
| ***MLL-AF4*** | TGGAGTAGGTCTGCTTTTCTTT |
| | TAGGTCTGCTTTTCTTTTGGTT * |
| ***MCL1*** | TTACACATCAATTCGTTCTGTA |
| | TGAAACTGAACTTTGCTTCTTT * |
| ***DUX4-IGH*** | TTCTGAAACCAAATCTGGACCC |
| | TTCGATTCTGAAACCAGATCTG * |
| ***DDIT4L*** | TTAGTTTGTTAGAACACTGGCT |
| | TAATATTTCTCATTTACTCTTA * |

* For each target an additional shRNA sequence was tested.

## Appendix to Supplementary Table 3.

Sequence of the 110bps oligo to be cloned into the pCDH-plasmid digested with XhoI and EcoRI enzymes:

XhoI passanger strand guide strand EcoRI

TCGAG**AAGGTATATTGCTGTTGACAGTGAGCG**CAAGAAAAGCAGACCTACTCCA**TAGTGAAGCCACAGATGTA**TGGAGTAGGTCTGCTTTTCTTT**TGCCTACTGCCTCG**G

5‘ common flank loop MLL/AF4 shRNA sequence 3‘ common flank

**Supplementary Table 4. Primers for qPCR**

| target | sequence |
|---|---|
| *HPRT1* | FW_TGATAGATCCATTCCTATGACTGTAGA |
| | RV_ CAAGACATTCTTTCCAGTTAAAGTTG |
| *MLL-AF4* | FW_AAGTTCCCAAAACCACTCCTAGT |
| | RV_GCCATGAATGGGTCATTTCC |
| *MLL* | FW_AAGTTCCCAAAACCACTCCTAGT |
| | RV_GATCCTGTGGACTCCATCTGC |
| *AF4* | FW_CTCCCCTCAAAAAGTGTTGC |
| | RV_TAGGTCTGCTCAACTGACTGAG |
| *DDIT4L* | FW_CCCAGAGAGCCTGCTAAGTG |
| | RV_TTGCTTTGATTTGGACAGACA |

# Supplementary Figures

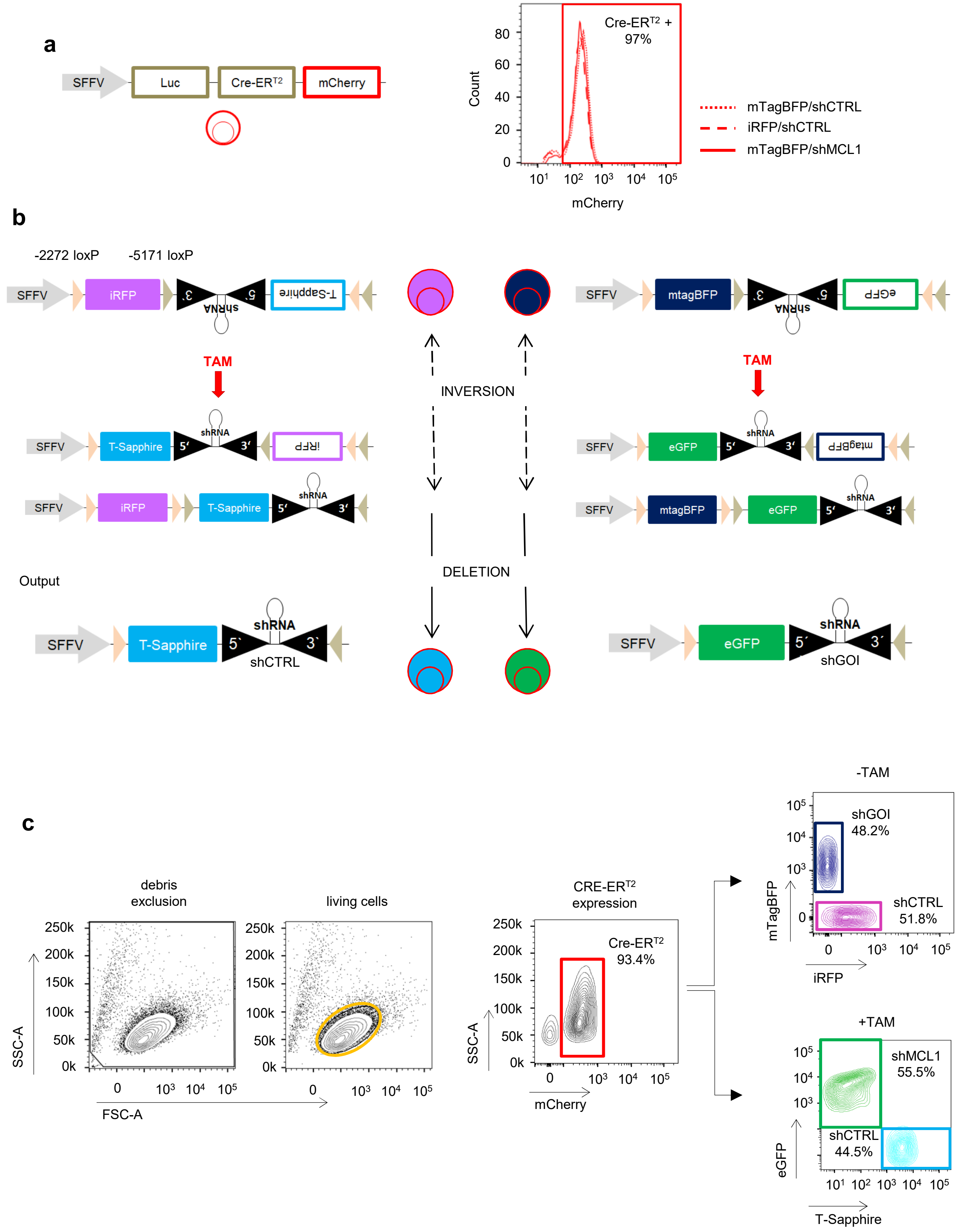

d

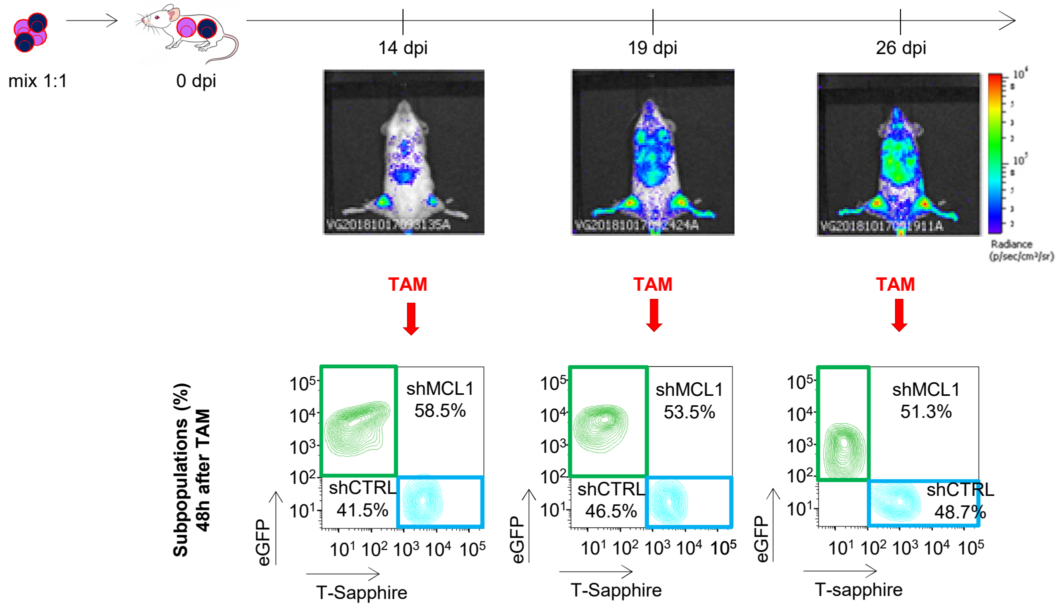

mix 1:1
0 dpi
14 dpi
19 dpi
26 dpi
Radiance (p/sec/cm²/sr)
TAM
TAM
TAM
Subpopulations (%) 48h after TAM
shMCL1 58.5%
shCTRL 41.5%
shMCL1 53.5%
shCTRL 46.5%
shMCL1 51.3%
shCTRL 48.7%
eGFP
T-Sapphire
T-Sapphire
T-sapphire


e

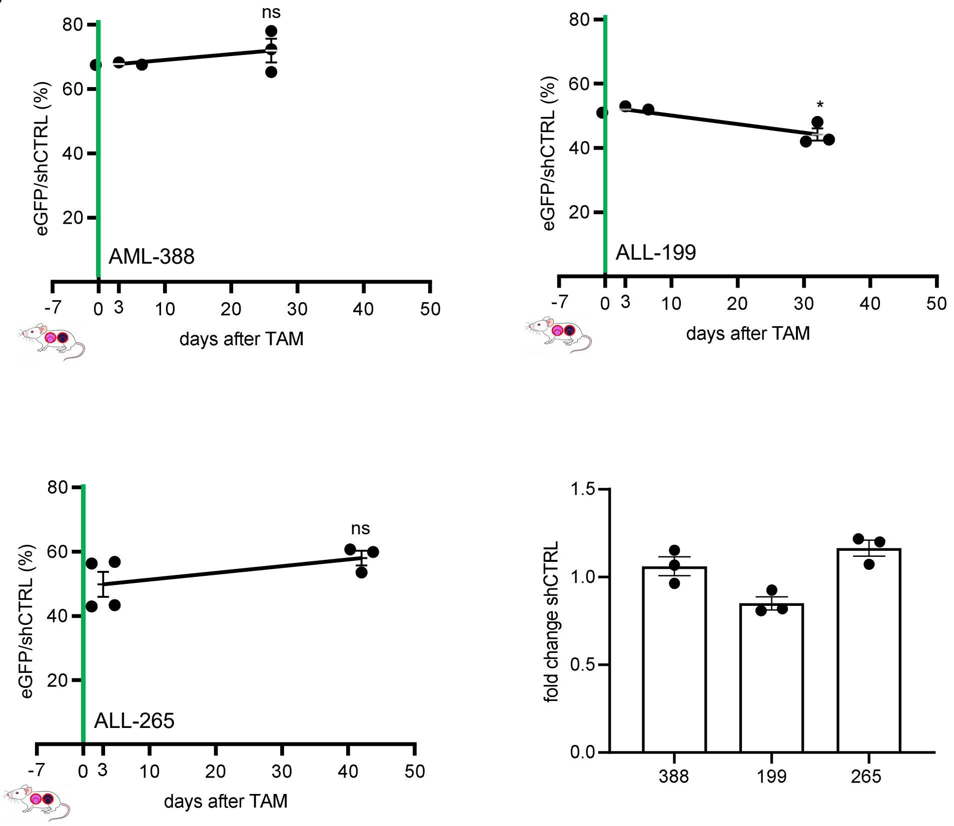

ns
eGFP/shCTRL (%)
AML-388
days after TAM
*
ALL-199
ns
ALL-265
fold change shCTRL
388
199
265

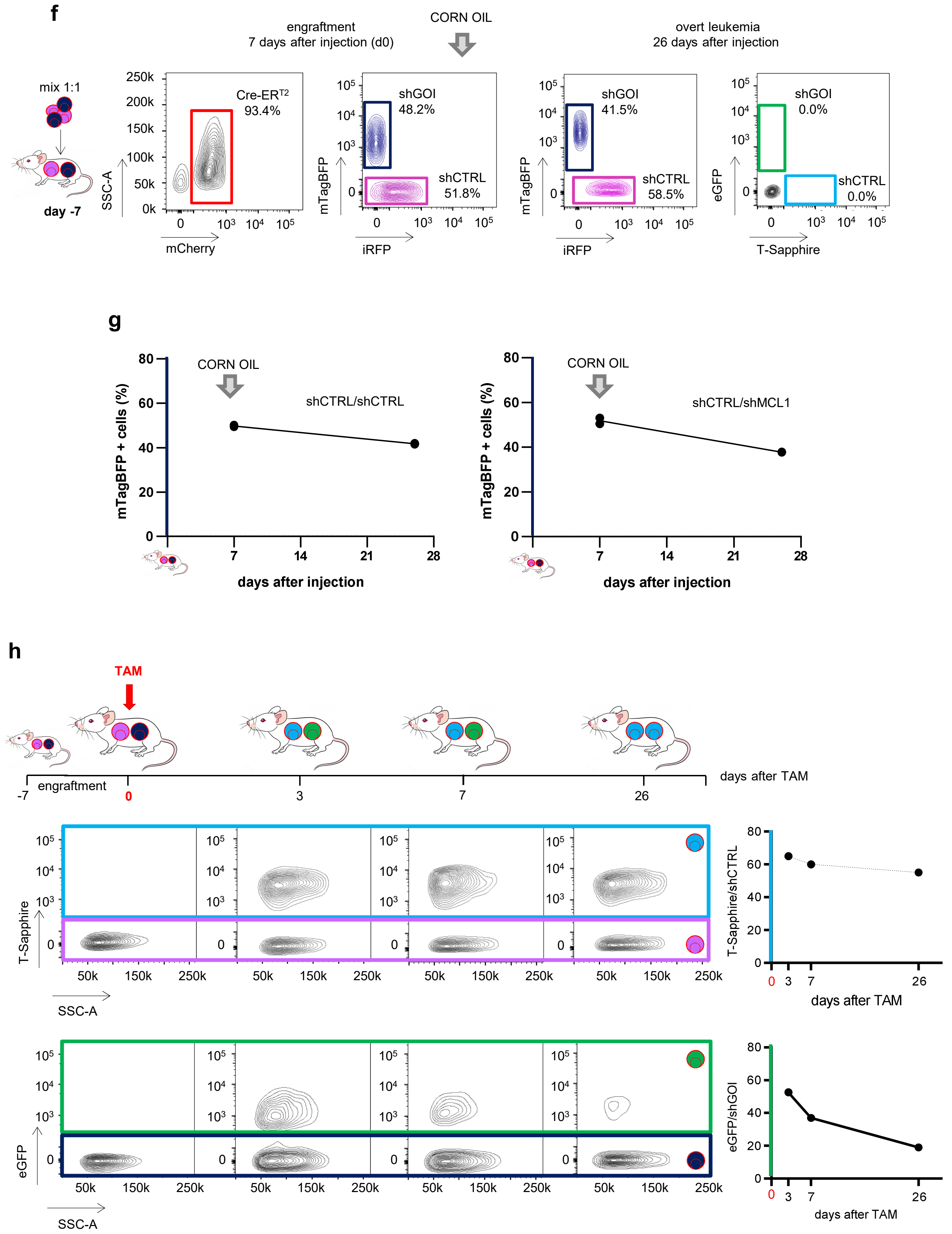


Suppl. Figure 1

**Supplementary Figure 1: Inducible knockdown system in PDX acute leukemia models in vivo and quality controls**

**a)** Details of the Cre-$ER^{T2}$ expression construct (left). Expression of a Gaussia luciferase (Luc) for in vivo imaging, Cre-$ER^{T2}$ and mCherry are under the control the SFFV promoter and connected via 2A-peptides. Histogram (right) displays expression levels of mCherry in different AML-388 PDX derivatives, co-transduced with different knockdown constructs; similar data were obtained in all 5 PDX models studied.
**b)** The 2-steps process of Cre-$ER^{T2}$ mediated recombination. The shRNA cassette is flanked by two different pairs of loxP sites; upon treatment of mice with TAM, Cre-$ER^{T2}$ translocates to the nucleus and first induces a reversible inversion between either of the two pairs of loxP sites (one example is shown); this converts the out-of-frame cassette into frame so that both, the inducible fluorochrome (T-Sapphire or eGFP) and the coupled shRNA, get under control of the SFFV promoter. In a second step, Cre-$ER^{T2}$ mediates an irreversible deletion between the second pair of loxP sites, resulting in deletion of the original fluorochrome (iRFP or mTagBFP). As end product, the constitutively expressed fluorochrome is lost, while the inducible fluorochrome is expressed in equimolar amounts together with the shRNA.
**c**) Gating strategy for the analysis of the competitive in vivo assays. All in vivo experiments have been analyzed following the depicted gating strategy. Debris exclusion, living cells gating (SSC-A/FSC-A), mCherry gating to analyze exclusively PDX cells expressing the CreERT2 enzyme. As last step, cells have been analyzed for the expression of mTagBFP or iRFP in the absence of TAM; or for the expression of eGFP or T-Sapphire after TAM administration.
**d)** Recombination efficiency is independent from tumor burden. Mice were injected with a mix of shCTRL/sh*MCL1* cells. Tumor growth was monitored by in vivo imaging; at the indicated time points, TAM was administered at 50 mg/kg per mouse to induce Cre-$ER^{T2}$-mediated inversion/deletion and consequent shRNA expression. Recombination efficiency was analyzed 48h after TAM by quantifying expression of the inducible fluorochrome markers by flow cytometry. Data from representative mice are displayed; 2 mice per time point were analyzed.
**e-g)** Quality control experiments:
e) Competitive in vivo experiments were set up as described in Figure 1c. The shCTRL/shCTRL mixture of AML-388 (n=6, $3*10^5$ cells/mouse), ALL-199 (n=6, $3*10^5$ cells/mouse) and ALL-265 (n=6, $3*10^5$ cells/mouse) was injected and eGFP-positive cells among all recombined cells were quantified at the indicated time points. To determine significance of depletion of eGFP-expressing cells, the percentage of eGFP cells at the experimental endpoint is compared to the percentage of eGFP cells at 3d post TAM. Mean ± SEM, * p=0.0185, ns not significant by unpaired t-test.
f) Competitive in vivo experiments were set up as described in Figure 1**c**, except that mice were injected with the solvent corn oil alone without TAM (n=4). One week after injection (day 0) two mice were sacrificed; flow cytometry plots show results from one representative mouse per time point; percentage of iRFP/shCTRL positive versus mTagBFP/shGOI (sh*MCL1*) positive cells was determined from all mCherry-Cre-$ER^{T2}$-positive cells. Corn oil was administered to the remaining two mice and cells analyzed 26 days after by flow cytometry.
g) Percentage of mTagBFP positive cells was quantified from all isolated cells from the experiment described in Figure S1e, expressing either mTagBFP or iRFP, for the two different mixtures shCTRL/shCTRL or shCTRL/sh*MCL1*.
h) Data complementing Figure 1c; from the shCTRL/sh*MCL1* mixture, shCTRL cells and sh*MCL1* cells were analyzed separately and not as pairwise competitive analysis as in Figure 1c. Upper row shows cells harboring the iRFP/shCTRL construct without shCTRL expression converting upon TAM treatment into T-Sapphire/shCTRL with shCTRL expression; lower row shows cells harboring the mTagBFP/shMCL1 construct without shMCL1 expression converting into eGFP/sh*MCL1* with sh*MCL1* expression. Right panels show quantification as [eGFP/shGOI positive cells divided by (the sum of mTagBFP/shGOI positive plus eGFP/shGOI positive cells)], respectively. The reliability of this type of analysis is restricted to settings with low cell death within the first 3 days.

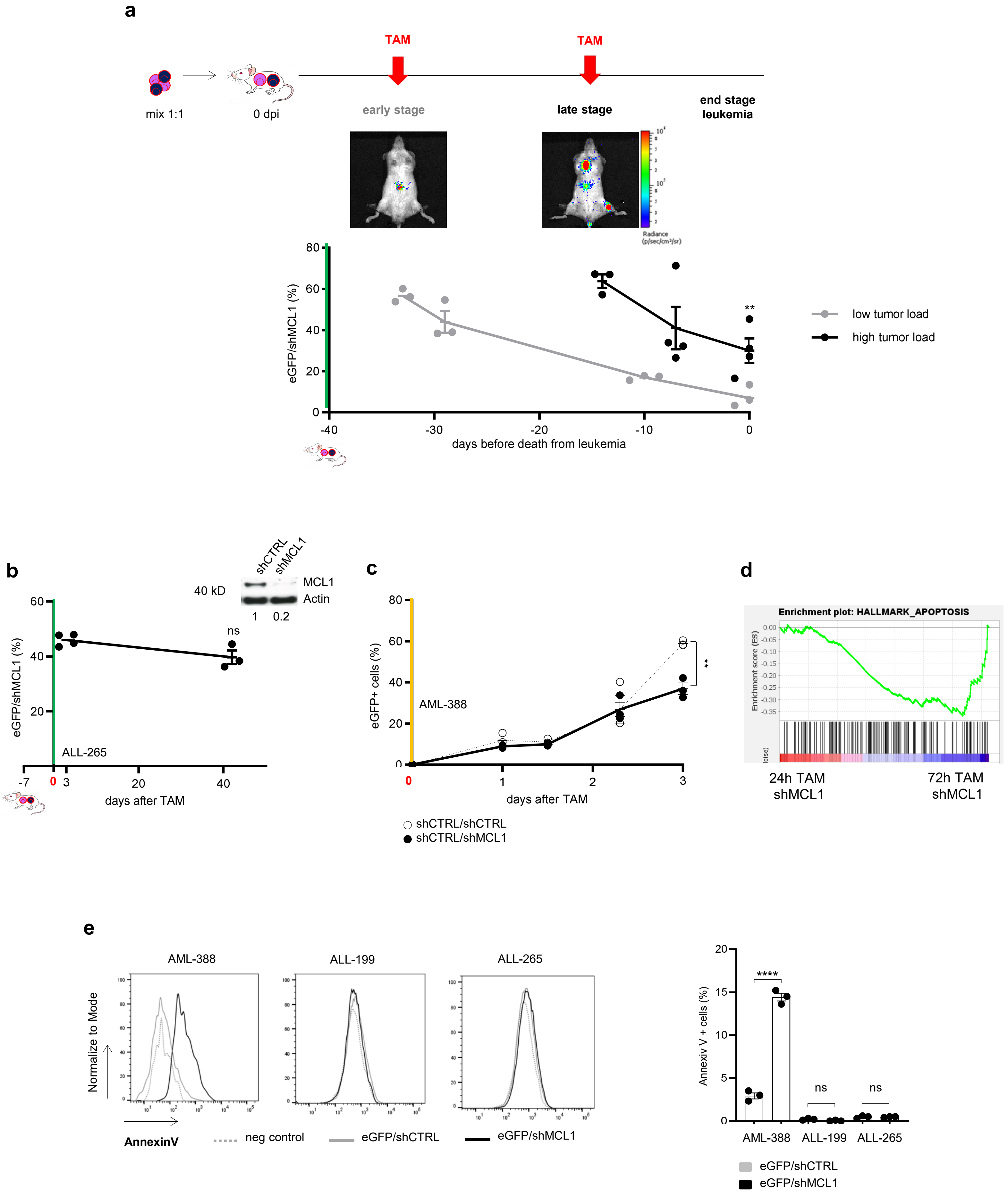


**Suppl. Figure 2**

**f**

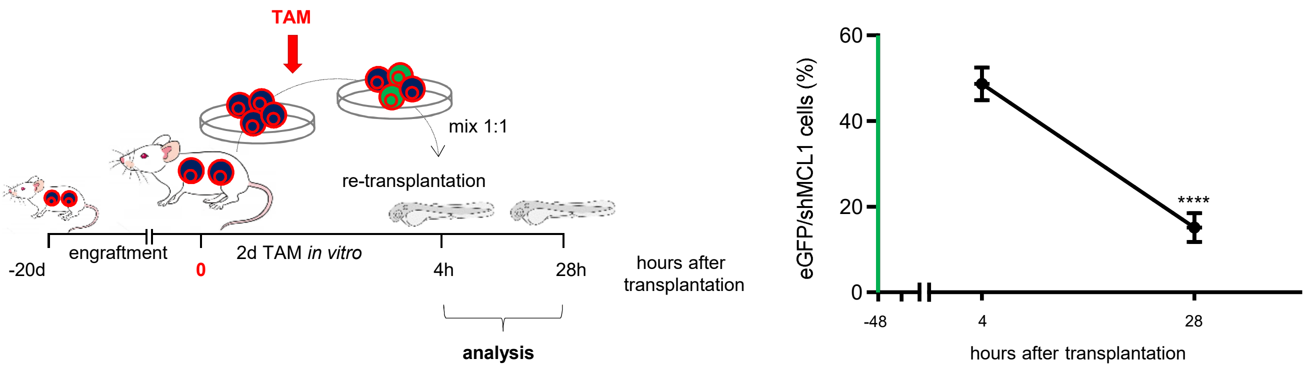


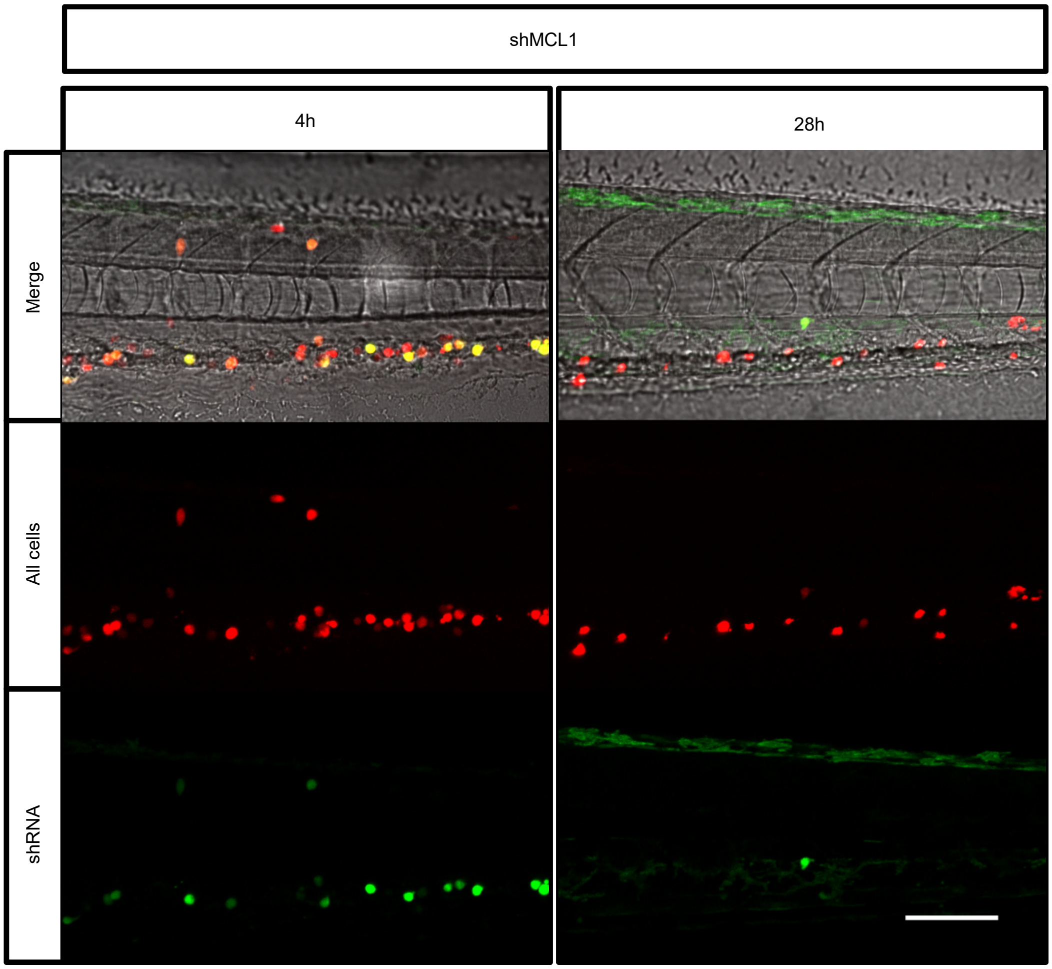


**g**

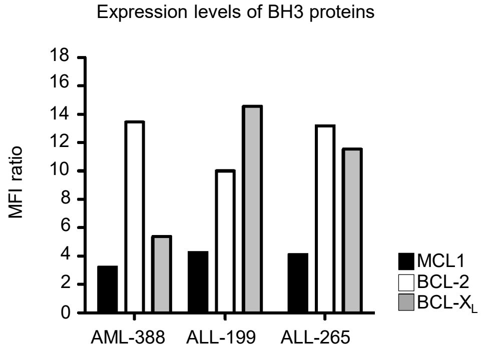


**Suppl. Figure 2**

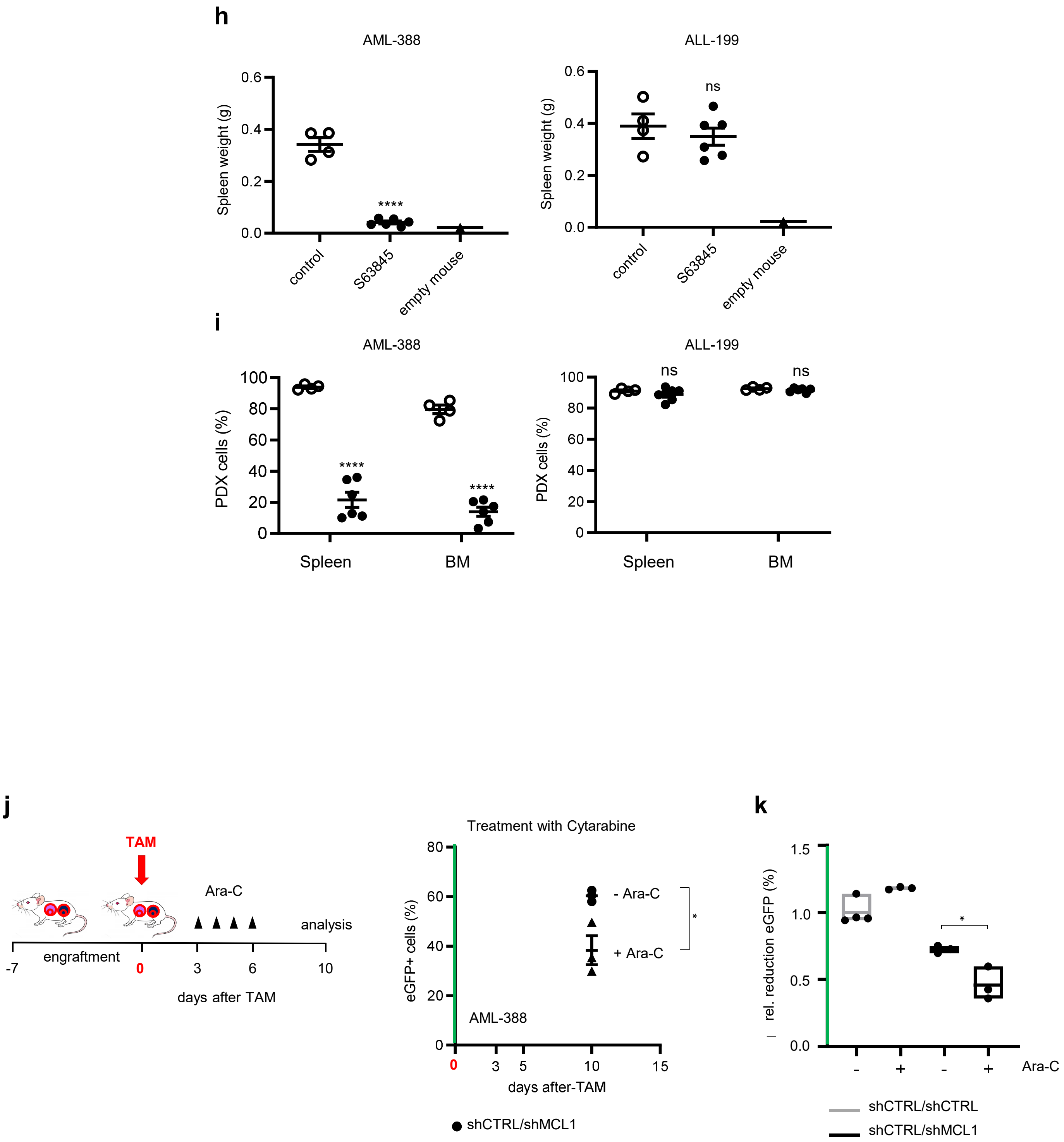


**Suppl. Figure 2**

**Supplementary Figure 2: *MCL1* is essential in AML-388 but dispensable in two ALL-PDX**

**a)** *MCL1* essentiality is independent from tumor burden. Experiments were set up as described in Figure 2b, except that 2*$10^6$ cells of the AML-388 shCTRL/shMCL1 mixture were injected and TAM was administered at a higher tumor burden. Mice were sacrificed 3 (n=3) and 10 (n=4) days after TAM and at end stage leukemia (n=4). Representative bioluminescence imaging pictures at the day of TAM administrations following injection of 3*$10^5$ (early stage; Figure 2b) and 2*$10^6$ (late stage) cells are shown. Graph displays mean ± SEM of the proportion of eGFP-positive cells isolated out of all recombined cells; grey line indicates results displayed in Figure 2b for comparison. Each dot represents one mouse. ** p=0.0067 by unpaired t-test.
**b)** Experiment described in Figure 2b was identically performed and depicted in ALL-265 (injection of 3*$10^5$ cells/mouse, n=7. Mean ± SEM of results is shown. At the end of the experiment, MCL1 protein expression was analyzed in sorted shCTRL and sh*MCL1* populations by Western blot (ALL-256). ns not significant by unpaired t-test.
**c)** *MCL1* knockdown cells are depleted early after TAM induction. For a kinetic of eGFP-expression at early time points after TAM, competitive experiments were performed and each subpopulation analyzed separately as described in Figure S1g; mice were analyzed at 24, 36, 52 and 72 hours after TAM administration (n=3 each). The analysis shows quantification as [eGFP/sh*MCL1* positive cells divided by (the sum of mTagBFP/sh*MCL1*-positive plus eGFP/sh*MCL1*-positive cells)]. The same analysis was performed for the shCTRL/shCTRL mixture. Mean ± SEM per group per time point is displayed. ** p=0.0016 by unpaired t-test.
**d)** Gene set enrichment analysis (GSEA) of transcriptome data from cells of experiment in Figure S2e, isolated 24 and 72 hours after TAM and sorted for eGFP/sh*MCL1* (n=3 per time point). NES= -1.52, P=0.0.
**e)** Knockdown of *MCL1* induces apoptosis; Annexin V staining in PDX AML-388, ALL-199 and ALL-265 3d after TAM. Representative histograms of 3 experiments are shown. Quantification of Annexin V positive eGFP/shCTRL or eGFP/sh*MCL1* cells (%) in PDX samples. Mean ± SEM of 3 independent experiments, **** p≤0.0001 by unpaired t-test.
**f)** Zebrafish experiment. Experimental layout: mCherry-Cre-ERT2 positive PDX cells from donor mice injected with AML-388 mTagBFP/sh*MCL1* cells were isolated from the BM of mice 20 days after injection. Cells were treated *ex vivo* with 50 nM TAM to induce eGFP/shRNA expression. After 48 hours, PDX cells were sorted to adjust cells with (eGFP positive) and without recombination (mTagBFP positive) to a 1:1 ratio. Cells were re-transplanted into groups of zebrafish embryos at 48 hours after fertilization (200 to 500 PDX cells per embryo). 4 (n=19) and 28 (n=18) hours after transplantation (hpt) (52 h and 76 h after TAM, respectively), larvae were anesthetized, and a field of view of the caudal hematopoietic tissue of each larvae was imaged to quantify mCherry and eGFP-positive cells. Graph displays mean ± SEM of the percentage of eGFP/sh*MCL1* positive cells among all transplanted, mCherry positive cells. **** p≤0.0001 by unpaired t-test with Welch's correction. Representative images of injected larvae with eGFP/sh*MCL1* expressing cells are displayed. Upper panel depicts merged images of the brightfield shot for anatomic orientation; mCherry positive cells are shown in red in the middle panel and eGFP/shRNA positive cells are shown in green in lower panel. Scale bar 100µm.
**g)** Intracellular expression levels of MCL1, BLC-2 and BCL-$X_L$, as measured by flow cytometry in the indicated PDX samples. Protein expression was calculated as the ratio of stained antibody mean fluorescent intensity (MFI) divided by isotype control MFI.
**h-i)** Pharmacological inhibition of MCL1. Mice from experiments in Figure 2d-f (n=4 for CTRL and n=6 for MCL1 inhibitor) were analyzed for **(h)** spleen weight and **(i)** the percentage of PDX cells among all cells isolated from spleen and bone marrow. Mean ± SEM , **** p ≤ 0.0001, ns not significant by unpaired t-test.
**j-k)** Combination treatment. Experiments were set up and analyzed as described in Figure 2g except that Cytarabine (Ara-C, 100 mg/kg/per day i.p. for 4 consecutive days, n=3) or solvent (n=3) was used. At the end of the experiment (10 days after TAM), mice were analyzed as in Figure 1c.
**j)** Mean ± SEM of 3 replicates per group and condition are shown. *p ≤ 0.0221 by unpaired t-test.
**k)** Reduction of eGFP-positive cells in the shCTRL/shMCL1 mix relative to shCTRL/shCTRL (+/- Ara-C) is displayed. Each dot represents one mouse. Mean ± SEM, *p ≤ 0.0221 by unpaired t-test.

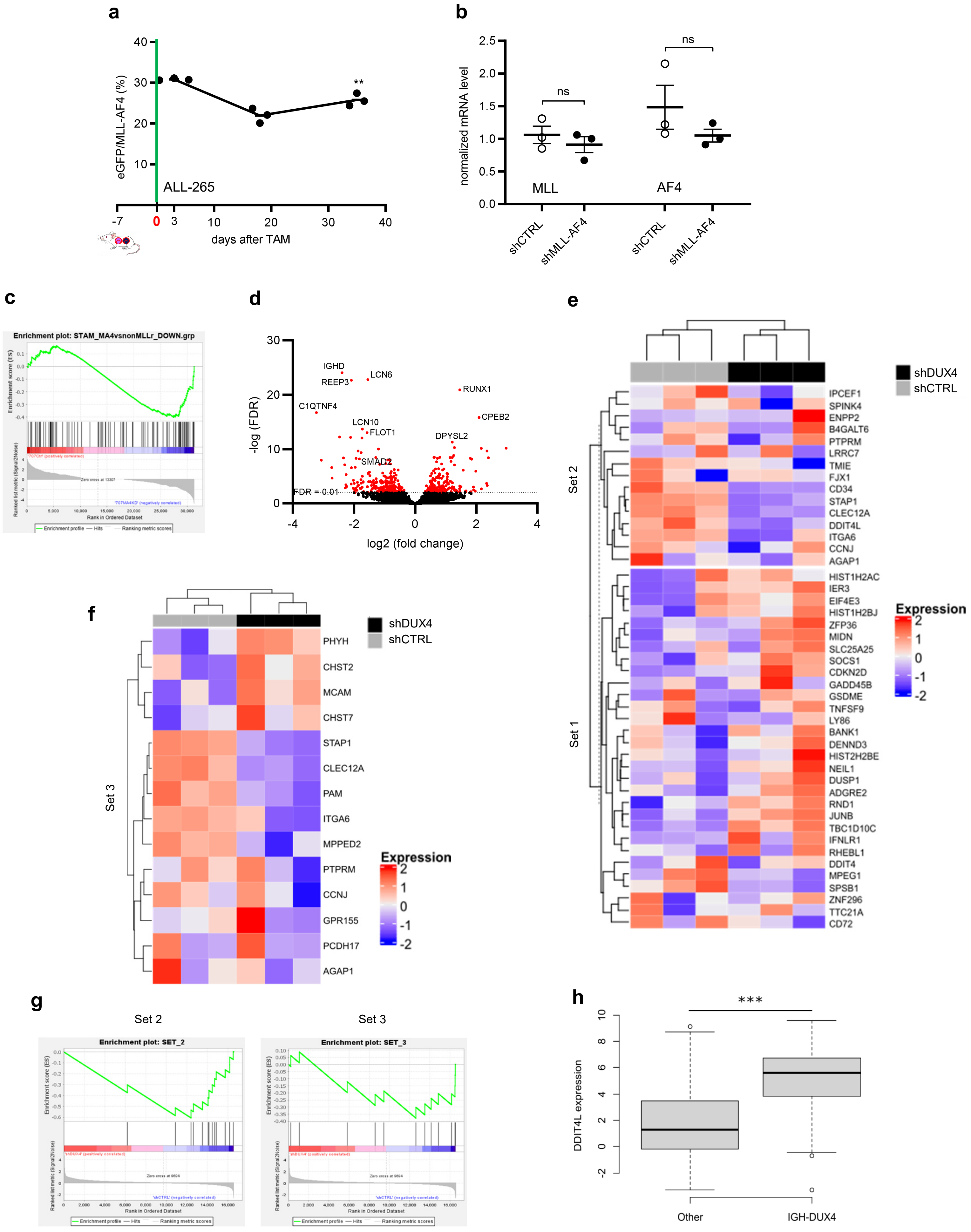


**Suppl. Figure 3**

**Supplementary Figure 3: In vivo functional validation of essential fusion genes**

**a-d)** Selective effects of sh*MLL-AF4*.
**a)** Experiments described in Figures 3a-b were performed using the non *MLL-AF4* rearranged ALL-265 PDX as control; TAM was applied once (50 mg/kg) 7 days post injections (day 0). Mice were sacrificed 3, 19 and 35 days after TAM (n=3 each). Percentage of eGFP-positive cells among all recombined cells was analyzed. Each dot represents one mouse. Mean ± SEM, ** p=0.0048 unpaired t-test.
**b)** mRNA expression of MLL and AF4 was analyzed by qPCR in ALL-265 cells expressing shCTRL or sh*MLL-AF4*. Mean ± SEM of cells isolated from n=3 mice, 35 days after TAM are shown. ns not significant by Welch's t-test.
**c)** Gene set enrichment analysis (GSEA) comparing an established *MLL-AF4* signature[1] with transcriptome data from cells of experiment in Figure 3b, isolated 28 days after TAM and sorted for eGFP/sh*MLL-AF4* and eGFP/shCTRL (n=3 per time point). NES= -1,37, P =0,031.
**d)** Differential expressed genes obtained from transcriptome data from experiment in Figure 3b are depicted as volcano blot (n=3). Genes with a high fold change are highly expressed shCTRL cells and low expressed in sh*MLL-AF4* cells.
**e-h)** Identification of therapeutic targets in DUX4-rearranged ALL. GSEA with two published datasets, Tanaka et al. 2018[2] and Harvey et al 2010[3]. Of 65 significant targets with a fold change of 2 in the Tanaka et al. dataset, 45 were present in our transcriptome dataset. To apply GSEA, targets were divided into upregulated (set 1) and downregulated (set 2) gene sets in *DUX4* knockdown cells. Of the Harvey et al dataset, which identified a transcriptome signature of pediatric B-precursor ALL patient samples with intragenic ERG deletions, 14 genes were present in our dataset (set 3).
**e)** Heatmap displaying expression of set 1 and set 2 between shCTRL and sh*DUX4*. All genes have been scaled to have the mean value of 0 and variance of 1.
**f)** Heatmap displaying expression of set 3 between shCTRL and sh*DUX4*. All genes have been scaled as described in Supplementary Figure 2e.

**g)** Enrichment plots for set 2 and 3. NES = -2.72 and -1.65, FDR q-value < 0.001 and q = 0.030, respectively.
**h)** *DDIT4L* expression values of 86 patients with an *IGH-DUX4* fusion were compared to patients without the fusion. *** p < 0.001 two-sided t-test.

## Supplementary References


1. Stam, R.W. et al. Gene expression profiling-based dissection of MLL translocated and MLL germline acute lymphoblastic leukemia in infants. *Blood* **115**, 2835-2844 (2010).
2. Tanaka, Y. et al. Transcriptional activities of DUX4 fusions in B-cell acute lymphoblastic leukemia. *Haematologica* **103**, e522-e526 (2018).
3. Harvey, R.C. et al. Identification of novel cluster groups in pediatric high-risk B-precursor acute lymphoblastic leukemia with gene expression profiling: correlation with genome-wide DNA copy number alterations, clinical characteristics, and outcome. *Blood* **116**, 4874-4884 (2010).
