# Supplementary material for: In vivo inducible reverse genetics in patients’ tumors to identify individual therapeutic targets
Source: Nat Commun. 2021 Sep 27;12:5655. doi: 10.1038/s41467-021-25963-z (PMC8476619; doi:10.1038/s41467-021-25963-z)
Supplement: Supplementary file 3 — Reporting summary [file 41467_2021_25963_MOESM3_ESM.pdf]

nature research

Corresponding author(s): Irmela Jeremias

Last updated by author(s): 2021-09-06

Reporting Summary

Nature Research wishes to improve the reproducibility of the work that we publish. This form provides structure for consistency and transparency in reporting. For further information on Nature Research policies, see our Editorial Policies and the Editorial Policy Checklist.

Statistics

For all statistical analyses, confirm that the following items are present in the figure legend, table legend, main text, or Methods section.

n/a	Confirmed	
☐	☒	The exact sample size (*n*) for each experimental group/condition, given as a discrete number and unit of measurement
☐	☒	A statement on whether measurements were taken from distinct samples or whether the same sample was measured repeatedly
☐	☒	The statistical test(s) used AND whether they are one- or two-sided<br>*Only common tests should be described solely by name; describe more complex techniques in the Methods section.*
☒	☐	A description of all covariates tested
☐	☒	A description of any assumptions or corrections, such as tests of normality and adjustment for multiple comparisons
☐	☒	A full description of the statistical parameters including central tendency (e.g. means) or other basic estimates (e.g. regression coefficient) AND variation (e.g. standard deviation) or associated estimates of uncertainty (e.g. confidence intervals)
☒	☐	For null hypothesis testing, the test statistic (e.g. *F*, *t*, *r*) with confidence intervals, effect sizes, degrees of freedom and *P* value noted<br>*Give P values as exact values whenever suitable.*
☒	☐	For Bayesian analysis, information on the choice of priors and Markov chain Monte Carlo settings
☒	☐	For hierarchical and complex designs, identification of the appropriate level for tests and full reporting of outcomes
☒	☐	Estimates of effect sizes (e.g. Cohen's *d*, Pearson's *r*), indicating how they were calculated

Our web collection on statistics for biologists contains articles on many of the points above.

Software and code

Policy information about availability of computer code

Data collection	BD FACSDiva 8.0.2 software was used to collect data from LSRII and FACS ARIA III
Data analysis	FlowJo 10.6.0 was used to analyze flow cytometry data; GraphPad Prism 8 for statistical analysis; Living Image software 4.4 (Caliper Life Sciences, Mainz, Germany) was used for data acquisition and quantification of light emission for in vivo imaging; for SCRB-seq data see detailed information in the Methods section.

For manuscripts utilizing custom algorithms or software that are central to the research but not yet described in published literature, software must be made available to editors and reviewers. We strongly encourage code deposition in a community repository (e.g. GitHub). See the Nature Research guidelines for submitting code & software for further information.

Data

Policy information about availability of data

All manuscripts must include a data availability statement. This statement should provide the following information, where applicable:

- Accession codes, unique identifiers, or web links for publicly available datasets
- A list of figures that have associated raw data
- A description of any restrictions on data availability

The RNA-seq data generated in this study have been deposited at the Gene Expression Omnibus under the following accession codes: GSE182760 (MCL1), GSE181973 (MLL-AF4), GSE182780 (DUX4-GH). Source data are provided with this paper.

Field-specific reporting

Life sciences study design

All studies must disclose on these points even when the disclosure is negative.

Sample size	Samples size was determined based on the variability observed between the different groups of animals, in preliminary control experiments. As SEM was very low, we could use 3/4 mice per group.
Data exclusions	No data were excluded from the analyses.
Replication	All in vivo experiments have been performed at least twice, with 3/4 mice each time point. We observed high reproducibility of the results.
Randomization	Allocation of animals to the different groups was random: indipendent from sex, cage, done by different investigators.
Blinding	Investigators were blind for data collection and analysis. Nevertheless blinding is not required.

Reporting for specific materials, systems and methods

We require information from authors about some types of materials, experimental systems and methods used in many studies. Here, indicate whether each material, system or method listed is relevant to your study. If you are not sure if a list item applies to your research, read the appropriate section before selecting a response.

Materials & experimental systems

n/a	Involved in the study
☐	☒ Antibodies
☒	☐ Eukaryotic cell lines
☒	☐ Palaeontology and archaeology
☐	☒ Animals and other organisms
☐	☒ Human research participants
☒	☐ Clinical data
☒	☐ Dual use research of concern

Methods

n/a	Involved in the study
☒	☐ ChIP-seq
☐	☒ Flow cytometry
☒	☐ MRI-based neuroimaging

Antibodies

Antibodies used	BCL-2 (clone Bcl-2/100, BD Bioscience), BCL-XL (clone 54H11, Cell Signaling, Cambridge, UK), MCL-1 (Clone D2W9E, Cell signaling) or respective isotype controls (Cat.: 556357, BD Bioscience; clone DA1E, Cell Signaling) for BH3 profiling. MCL1 (D3CA5, Cell Signaling Technologies), DUX4 (MAB9535, R&D system) and β-actin (NB600-501SS, Novus Biologicals) for immunoassays. MCL1 (S-19, Santa Cruz Biotechnology) and GAPDH (6C5, Merck Millipore) for the Western Blot of PDX-265.
Validation	Validation of the antibodies used for the immunoassay analyses was performed by the company : Simple Western protein immunoassay (WES, ProteinSimple, San Jose, USA). BD Bioscience and Cell signaling fluorescently labeled antibodies for flow cytometry: In order to ensure consistently high-quality reagents, each lot of antibody is tested and validated by the company. Representative flow cytometry data are always included in the technical data sheets, together with citations of articles where the specific antibody was used. Western Blot antibodies from Santa Cruz Biotechnology and Merck Millipore have been tested by the company. Related Western Blot's images can be found online.

Animals and other organisms

Policy information about studies involving animals; ARRIVE guidelines recommended for reporting animal research

Laboratory animals	NOD scid gamma (NSG) mice, male animals were used in the presence of TAM when possible. Animals were older than 6 weeks. Danio rerio, AB line, 48h after fertilization
Wild animals	No wild animals were used in this study.
Field-collected samples	No field-collected samples were used in this study.
Ethics oversight	Written approval by Regierung von Oberbayern, Az. ROB55.2Vet-2532.Vet02-16-7; Az. ROB-55.2Vet-2532.Vet02- 15-193; Az. ROB-55.2Vet-2532.Vet03-16-56;

Note that full information on the approval of the study protocol must also be provided in the manuscript.

Flow Cytometry

Plots

Confirm that:

- [x] The axis labels state the marker and fluorochrome used (e.g. CD4-FITC).
- [x] The axis scales are clearly visible. Include numbers along axes only for bottom left plot of group (a 'group' is an analysis of identical markers).
- [x] All plots are contour plots with outliers or pseudocolor plots.
- [x] A numerical value for number of cells or percentage (with statistics) is provided.

Methodology

Sample preparation	PDX cells used in this work were isolated from the BM of the animals using a mortar. Washed twice in PBS and counted.
Instrument	Ceramic mortar
Software	FlowJo was used to analyse all flow cytometry data.
Cell population abundance	A minimum of 5000 events was analyzed for each samples using the LSRII.<br>Cell populations were purified using a FACS ARIA. Purity > 94%.
Gating strategy	The gating strategy is described in details in text and Supplemental Figures.

- [x] Tick this box to confirm that a figure exemplifying the gating strategy is provided in the Supplementary Information.

All in vivo experiments have been gated as follow:
FSC/SSC >> Cre-ERT2-mCherry positive cells (easy to set the gate for positivity, as NEG and POS populations were nicely apart in all samples)
> mtagBFP/iRPF to analyze subpopulations' distribution in the absence of TAM, eGFP/T-Sapphire to analyze subpopulations' distribution after TAM administration (recombined cells). All different color populations could be easily distinguished from each other, with no problem for setting the gate for positivity.
